# Bis[(*E*)-4-chloro-2-(cyclo­hexyl­imino­meth­yl)phenolato]nickel(II)

**DOI:** 10.1107/S1600536808015390

**Published:** 2008-05-30

**Authors:** Dong-Sheng Xia, Wu Chen, Yu-Min Zhao, Qing-Fu Zeng

**Affiliations:** a Engineering Research Center for the Clean Production of Textile Printing, Ministry of Education, Wuhan University of Science and Engineering, Wuhan 430073, People’s Republic of China

## Abstract

In the title mononuclear nickel(II) complex, [Ni(C_13_H_15_ClNO)_2_], the Ni^II^ atom is four-coordinated in a tetra­hedral geometry by the N and O atoms of the two Schiff base ligands.

## Related literature

For related structures, see: Cheng *et al.* (2007 ▶); Li *et al.* (2007 ▶); Qiu *et al.* (2006 ▶); Shi *et al.* (2007 ▶); Wang *et al.* (2005 ▶); Zhu *et al.* (2003 ▶).
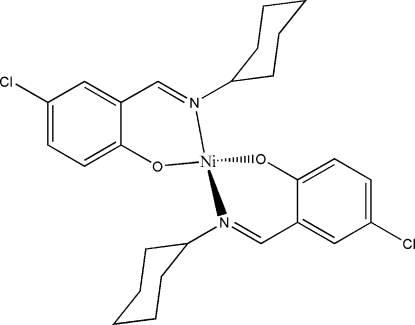


## Experimental

### 

#### Crystal data

[Ni(C_13_H_15_ClNO)_2_]
*M* *_r_* = 532.13
Orthorhombic, 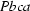

*a* = 14.871 (3) Å
*b* = 13.563 (3) Å
*c* = 24.993 (5) Å
*V* = 5040.9 (17) Å^3^
*Z* = 8
Mo *K*α radiation
μ = 1.01 mm^−1^
*T* = 298 (2) K
0.42 × 0.38 × 0.37 mm

#### Data collection

Enraf–Nonius CAD-4 diffractometer
Absorption correction: ψ scan (North *et al.*, 1968 ▶) *T*_min_ = 0.663, *T*_max_ = 0.686
4933 measured reflections
*R*_int_ = 0.023
4693 independent reflections
2416 reflections with *I* > 2σ(*I*)

#### Refinement

*R*[*F* ^2^ > 2σ(*F* ^2^)] = 0.066
*wR*(*F* ^2^) = 0.162
*S* = 1.04
4693 reflections
298 parameters
H-atom parameters constrained
Δρ_max_ = 0.32 e Å^−3^
Δρ_min_ = −0.45 e Å^−3^

### 

Data collection: *CAD-4 Software* (Enraf–Nonius, 1989 ▶); cell refinement: *CAD-4 Software*; data reduction: *XCAD4* (Harms & Wocadlo, 1995 ▶); program(s) used to solve structure: *SHELXS97* (Sheldrick, 2008 ▶); program(s) used to refine structure: *SHELXL97* (Sheldrick, 2008 ▶); molecular graphics: *SHELXTL* (Sheldrick, 2008 ▶); software used to prepare material for publication: *SHELXTL*.

## Supplementary Material

Crystal structure: contains datablocks global, I. DOI: 10.1107/S1600536808015390/sj2506sup1.cif

Structure factors: contains datablocks I. DOI: 10.1107/S1600536808015390/sj2506Isup2.hkl

Additional supplementary materials:  crystallographic information; 3D view; checkCIF report

## Figures and Tables

**Table:** 

Ni1—O1	1.911 (4)
Ni1—O2	1.911 (4)
Ni1—N1	2.016 (5)
Ni1—N2	2.018 (5)

**Table:** 

O1—Ni1—O2	120.45 (18)
O1—Ni1—N1	95.93 (18)
O2—Ni1—N1	112.44 (18)
O1—Ni1—N2	113.09 (19)
O2—Ni1—N2	94.54 (17)
N1—Ni1—N2	122.42 (18)

**Fig. 1.**
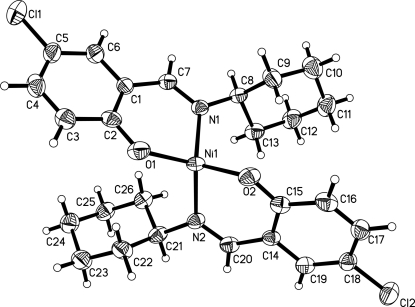
The structure of (I) showing 30% probability displacement ellipsoids and the atom-numbering scheme.

## supplementary crystallographic information

### Comment

As part of our ongoing interest in the structure of nickel(II) complexes (Zhu *et al.*, 2003), we report herein the crystal structure of the title compound, a new mononuclear nickel(II) complex, (I), Fig. 1, derived from the Schiff base ligand 4-chloro-2-(cyclohexyliminomethyl)phenol.

The Ni^II^ atom in (I) is four-coordinate in a tetrahedral geometry, binding to the N and two O atoms of the two Schiff base ligands. The coordinate bond values (Table 1) are comparable to values observed in other similar nickel(II) complexes (Shi *et al.*, 2007; Li *et al.*, 2007; Cheng *et al.*, 2007; Qiu *et al.*, 2006; Wang *et al.*, 2005).

### Experimental

5-Chlorosalicylaldehyde (15.6 mg, 0.1 mmol), cyclohexylamine (9.9 mg, 0.1 mmol), and NiCl_2_.6H_2_O (23.8 mg, 0.1 mmol) were dissolved in methanol (30 ml). The mixture was stirred for 30 min at room temperature. The resulting solution was left in air for a few days, yielding green crystals.

### Refinement

H atoms were placed in idealized positions and constrained to ride on their parent atoms with C–H distances in the range 0.93–0.98 Å, and with *U*_iso_(H) set at 1.2*U*_eq_(C).

### Crystal data

**Table:**

[Ni(C_13_H_15_ClNO)_2_]	*F*_000_ = 2224
*M**_r_* = 532.13	*D*_x_ = 1.402 Mg m^−^^3^
Orthorhombic, *P**b**c**a*	Mo *K*α radiation λ = 0.71073 Å
Hall symbol: -P 2ac 2ab	Cell parameters from 2013 reflections
*a* = 14.871 (3) Å	θ = 2.3–25.3º
*b* = 13.563 (3) Å	µ = 1.01 mm^−^^1^
*c* = 24.993 (5) Å	*T* = 298 (2) K
*V* = 5040.9 (17) Å^3^	Block, green
*Z* = 8	0.42 × 0.38 × 0.37 mm

### Data collection

**Table:**

Bruker SMART CCD area-detector diffractometer	4693 independent reflections
Radiation source: fine-focus sealed tube	2416 reflections with *I* > 2σ(*I*)
Monochromator: graphite	*R*_int_ = 0.023
*T* = 298(2) K	θ_max_ = 25.5º
ω scans	θ_min_ = 2.1º
Absorption correction: multi-scan(SADABS; Sheldrick, 1996)	*h* = −17→18
*T*_min_ = 0.663, *T*_max_ = 0.686	*k* = −15→16
4933 measured reflections	*l* = −30→30

### Refinement

**Table:**

Refinement on *F*^2^	Secondary atom site location: difference Fourier map
Least-squares matrix: full	Hydrogen site location: inferred from neighbouring sites
*R*[*F*^2^ > 2σ(*F*^2^)] = 0.066	H-atom parameters constrained
*wR*(*F*^2^) = 0.162	*w* = 1/[σ^2^(*F*_o_^2^) + (0.0617*P*)^2^] where *P* = (*F*_o_^2^ + 2*F*_c_^2^)/3
*S* = 1.04	(Δ/σ)_max_ = 0.001
4693 reflections	Δρ_max_ = 0.32 e Å^−^^3^
298 parameters	Δρ_min_ = −0.45 e Å^−^^3^
Primary atom site location: structure-invariant direct methods	Extinction correction: none

### Special details

**Table:**

Geometry. All esds (except the esd in the dihedral angle between two l.s. planes) are estimated using the full covariance matrix. The cell esds are taken into account individually in the estimation of esds in distances, angles and torsion angles; correlations between esds in cell parameters are only used when they are defined by crystal symmetry. An approximate (isotropic) treatment of cell esds is used for estimating esds involving l.s. planes.
Refinement. Refinement of F^2^ against ALL reflections. The weighted R-factor wR and goodness of fit S are based on F^2^, conventional R-factors R are based on F, with F set to zero for negative F^2^. The threshold expression of F^2^ > 2sigma(F^2^) is used only for calculating R-factors(gt) etc. and is not relevant to the choice of reflections for refinement. R-factors based on F^2^ are statistically about twice as large as those based on F, and R- factors based on ALL data will be even larger.

### Fractional atomic coordinates and isotropic or equivalent isotropic displacement parameters (Å^2^)

**Table:**

	*x*	*y*	*z*	*U*_iso_*/*U*_eq_	
Ni1	0.14197 (4)	0.91723 (5)	0.19419 (3)	0.0418 (2)	
Cl1	−0.11240 (16)	0.61620 (16)	0.01142 (8)	0.1021 (8)	
Cl2	0.32265 (12)	1.23468 (13)	0.39664 (7)	0.0768 (6)	
O1	0.1603 (3)	0.8322 (3)	0.13420 (17)	0.0644 (12)	
O2	0.2180 (3)	0.9080 (3)	0.25567 (16)	0.0586 (11)	
N1	0.0151 (3)	0.8732 (3)	0.20947 (18)	0.0460 (12)	
N2	0.1735 (3)	1.0591 (3)	0.17874 (19)	0.0482 (13)	
C1	0.0059 (4)	0.7796 (4)	0.1256 (2)	0.0473 (15)	
C2	0.0954 (4)	0.7884 (4)	0.1071 (3)	0.0524 (16)	
C3	0.1154 (4)	0.7475 (5)	0.0575 (3)	0.0618 (17)	
H3	0.1729	0.7560	0.0435	0.074*	
C4	0.0536 (5)	0.6954 (5)	0.0286 (3)	0.069 (2)	
H4	0.0699	0.6676	−0.0040	0.083*	
C5	−0.0337 (5)	0.6836 (5)	0.0478 (3)	0.0648 (19)	
C6	−0.0575 (4)	0.7255 (4)	0.0950 (2)	0.0586 (17)	
H6	−0.1162	0.7187	0.1075	0.070*	
C7	−0.0277 (4)	0.8180 (4)	0.1755 (2)	0.0501 (16)	
H7	−0.0865	0.8014	0.1844	0.060*	
C8	−0.0329 (4)	0.9005 (4)	0.2591 (2)	0.0486 (15)	
H8	−0.0970	0.8860	0.2545	0.058*	
C9	0.0027 (5)	0.8394 (5)	0.3045 (2)	0.0683 (19)	
H9A	0.0673	0.8485	0.3072	0.082*	
H9B	−0.0086	0.7703	0.2973	0.082*	
C10	−0.0411 (5)	0.8677 (5)	0.3573 (3)	0.083 (2)	
H10A	−0.1047	0.8520	0.3560	0.100*	
H10B	−0.0142	0.8298	0.3861	0.100*	
C11	−0.0291 (5)	0.9768 (6)	0.3682 (3)	0.079 (2)	
H11A	−0.0592	0.9944	0.4013	0.095*	
H11B	0.0344	0.9920	0.3721	0.095*	
C12	−0.0682 (5)	1.0352 (5)	0.3224 (3)	0.072 (2)	
H12A	−0.1320	1.0212	0.3195	0.087*	
H12B	−0.0612	1.1051	0.3295	0.087*	
C13	−0.0222 (4)	1.0095 (4)	0.2705 (3)	0.0577 (17)	
H13A	0.0412	1.0259	0.2728	0.069*	
H13B	−0.0483	1.0476	0.2416	0.069*	
C14	0.2385 (3)	1.0838 (4)	0.2672 (2)	0.0449 (14)	
C15	0.2402 (4)	0.9843 (5)	0.2852 (3)	0.0498 (16)	
C16	0.2693 (4)	0.9689 (5)	0.3380 (3)	0.0601 (18)	
H16	0.2719	0.9045	0.3507	0.072*	
C17	0.2941 (4)	1.0439 (5)	0.3720 (3)	0.0622 (18)	
H17	0.3118	1.0304	0.4069	0.075*	
C18	0.2920 (4)	1.1408 (5)	0.3530 (2)	0.0530 (16)	
C19	0.2665 (4)	1.1603 (4)	0.3020 (2)	0.0520 (16)	
H19	0.2674	1.2250	0.2897	0.062*	
C20	0.2118 (4)	1.1130 (4)	0.2144 (2)	0.0507 (15)	
H20	0.2236	1.1781	0.2049	0.061*	
C21	0.1556 (4)	1.1016 (4)	0.1257 (2)	0.0551 (17)	
H21	0.1659	1.1729	0.1275	0.066*	
C22	0.2191 (4)	1.0582 (5)	0.0844 (2)	0.0644 (19)	
H22A	0.2157	0.9868	0.0860	0.077*	
H22B	0.2802	1.0774	0.0931	0.077*	
C23	0.1970 (4)	1.0921 (6)	0.0278 (2)	0.076 (2)	
H23A	0.2083	1.1623	0.0247	0.092*	
H23B	0.2360	1.0584	0.0026	0.092*	
C24	0.0995 (4)	1.0709 (5)	0.0138 (3)	0.071 (2)	
H24A	0.0894	1.0002	0.0135	0.085*	
H24B	0.0863	1.0961	−0.0217	0.085*	
C25	0.0371 (4)	1.1194 (5)	0.0545 (3)	0.0648 (19)	
H25A	0.0444	1.1904	0.0530	0.078*	
H25B	−0.0249	1.1041	0.0457	0.078*	
C26	0.0575 (4)	1.0836 (5)	0.1107 (2)	0.0575 (16)	
H26A	0.0446	1.0136	0.1131	0.069*	
H26B	0.0189	1.1177	0.1359	0.069*	

### Atomic displacement parameters (Å^2^)

**Table:**

	*U*^11^	*U*^22^	*U*^33^	*U*^12^	*U*^13^	*U*^23^
Ni1	0.0369 (4)	0.0331 (4)	0.0554 (5)	−0.0024 (3)	0.0001 (4)	0.0037 (4)
Cl1	0.1222 (19)	0.1082 (17)	0.0760 (14)	−0.0257 (14)	−0.0106 (13)	−0.0268 (12)
Cl2	0.0907 (13)	0.0690 (12)	0.0706 (12)	−0.0022 (10)	−0.0220 (10)	−0.0051 (10)
O1	0.054 (3)	0.055 (3)	0.085 (3)	−0.005 (2)	0.012 (2)	−0.010 (2)
O2	0.064 (3)	0.038 (2)	0.074 (3)	−0.003 (2)	−0.008 (2)	0.006 (2)
N1	0.047 (3)	0.039 (3)	0.052 (3)	−0.002 (2)	0.007 (3)	−0.002 (2)
N2	0.045 (3)	0.045 (3)	0.055 (3)	−0.003 (2)	−0.012 (2)	0.006 (2)
C1	0.057 (4)	0.039 (3)	0.047 (4)	0.002 (3)	0.007 (3)	−0.001 (3)
C2	0.055 (4)	0.037 (3)	0.065 (5)	0.003 (3)	0.012 (4)	0.007 (3)
C3	0.064 (4)	0.056 (4)	0.066 (5)	0.008 (4)	0.013 (4)	−0.001 (4)
C4	0.097 (6)	0.055 (4)	0.055 (4)	0.016 (4)	0.011 (4)	−0.003 (4)
C5	0.090 (5)	0.051 (4)	0.054 (4)	0.000 (4)	0.000 (4)	−0.006 (3)
C6	0.064 (4)	0.059 (4)	0.053 (4)	0.000 (4)	−0.003 (4)	0.004 (3)
C7	0.045 (4)	0.046 (4)	0.059 (4)	−0.005 (3)	0.004 (3)	0.000 (3)
C8	0.044 (3)	0.045 (4)	0.057 (4)	−0.005 (3)	0.002 (3)	−0.003 (3)
C9	0.097 (5)	0.052 (4)	0.057 (4)	0.004 (4)	0.017 (4)	0.001 (4)
C10	0.107 (6)	0.074 (5)	0.070 (5)	0.006 (5)	0.011 (5)	−0.001 (4)
C11	0.085 (6)	0.087 (6)	0.064 (5)	0.000 (5)	0.005 (4)	−0.021 (5)
C12	0.081 (5)	0.059 (4)	0.077 (5)	0.002 (4)	0.005 (4)	−0.020 (4)
C13	0.057 (4)	0.043 (4)	0.073 (5)	0.004 (3)	0.009 (3)	−0.010 (3)
C14	0.038 (3)	0.043 (3)	0.054 (4)	−0.003 (3)	−0.005 (3)	0.010 (3)
C15	0.039 (3)	0.048 (4)	0.062 (4)	0.003 (3)	−0.001 (3)	0.008 (3)
C16	0.054 (4)	0.052 (4)	0.074 (5)	0.006 (3)	−0.007 (4)	0.026 (4)
C17	0.057 (4)	0.072 (5)	0.057 (4)	−0.001 (4)	−0.006 (3)	0.007 (4)
C18	0.049 (4)	0.056 (4)	0.054 (4)	0.000 (3)	−0.009 (3)	0.004 (3)
C19	0.046 (4)	0.048 (4)	0.062 (4)	0.001 (3)	−0.003 (3)	0.007 (4)
C20	0.045 (3)	0.044 (4)	0.063 (4)	0.000 (3)	−0.005 (3)	0.011 (3)
C21	0.059 (4)	0.039 (4)	0.067 (4)	−0.004 (3)	−0.019 (3)	0.006 (3)
C22	0.045 (4)	0.084 (5)	0.064 (4)	−0.004 (3)	−0.005 (3)	0.017 (4)
C23	0.063 (5)	0.099 (6)	0.067 (5)	−0.013 (4)	−0.007 (4)	0.010 (4)
C24	0.069 (5)	0.081 (5)	0.064 (5)	−0.004 (4)	−0.015 (4)	0.003 (4)
C25	0.063 (4)	0.060 (4)	0.072 (5)	0.001 (4)	−0.021 (4)	0.002 (4)
C26	0.053 (4)	0.053 (4)	0.066 (4)	0.004 (3)	−0.009 (3)	0.000 (4)

### Geometric parameters (Å, °)

**Table:**

Ni1—O1	1.911 (4)	C11—H11B	0.9700
Ni1—O2	1.911 (4)	C12—C13	1.508 (8)
Ni1—N1	2.016 (5)	C12—H12A	0.9700
Ni1—N2	2.018 (5)	C12—H12B	0.9700
Cl1—C5	1.740 (7)	C13—H13A	0.9700
Cl2—C18	1.737 (6)	C13—H13B	0.9700
O1—C2	1.321 (7)	C14—C19	1.417 (7)
O2—C15	1.314 (7)	C14—C15	1.423 (7)
N1—C7	1.298 (7)	C14—C20	1.434 (7)
N1—C8	1.478 (7)	C15—C16	1.404 (8)
N2—C20	1.286 (7)	C16—C17	1.374 (8)
N2—C21	1.470 (7)	C16—H16	0.9300
C1—C2	1.415 (8)	C17—C18	1.398 (8)
C1—C6	1.419 (8)	C17—H17	0.9300
C1—C7	1.440 (7)	C18—C19	1.356 (7)
C2—C3	1.390 (8)	C19—H19	0.9300
C3—C4	1.366 (8)	C20—H20	0.9300
C3—H3	0.9300	C21—C22	1.516 (8)
C4—C5	1.394 (9)	C21—C26	1.526 (7)
C4—H4	0.9300	C21—H21	0.9800
C5—C6	1.358 (8)	C22—C23	1.525 (8)
C6—H6	0.9300	C22—H22A	0.9700
C7—H7	0.9300	C22—H22B	0.9700
C8—C9	1.502 (8)	C23—C24	1.520 (8)
C8—C13	1.514 (7)	C23—H23A	0.9700
C8—H8	0.9800	C23—H23B	0.9700
C9—C10	1.520 (8)	C24—C25	1.527 (8)
C9—H9A	0.9700	C24—H24A	0.9700
C9—H9B	0.9700	C24—H24B	0.9700
C10—C11	1.516 (9)	C25—C26	1.516 (8)
C10—H10A	0.9700	C25—H25A	0.9700
C10—H10B	0.9700	C25—H25B	0.9700
C11—C12	1.508 (9)	C26—H26A	0.9700
C11—H11A	0.9700	C26—H26B	0.9700
			
O1—Ni1—O2	120.45 (18)	H12A—C12—H12B	108.0
O1—Ni1—N1	95.93 (18)	C12—C13—C8	109.9 (5)
O2—Ni1—N1	112.44 (18)	C12—C13—H13A	109.7
O1—Ni1—N2	113.09 (19)	C8—C13—H13A	109.7
O2—Ni1—N2	94.54 (17)	C12—C13—H13B	109.7
N1—Ni1—N2	122.42 (18)	C8—C13—H13B	109.7
C2—O1—Ni1	124.8 (4)	H13A—C13—H13B	108.2
C15—O2—Ni1	123.3 (4)	C19—C14—C15	119.7 (5)
C7—N1—C8	117.2 (5)	C19—C14—C20	116.4 (5)
C7—N1—Ni1	120.4 (4)	C15—C14—C20	124.0 (5)
C8—N1—Ni1	122.4 (4)	O2—C15—C16	119.3 (6)
C20—N2—C21	118.8 (5)	O2—C15—C14	124.4 (6)
C20—N2—Ni1	120.8 (4)	C16—C15—C14	116.3 (6)
C21—N2—Ni1	120.3 (4)	C17—C16—C15	123.6 (6)
C2—C1—C6	119.5 (6)	C17—C16—H16	118.2
C2—C1—C7	125.5 (6)	C15—C16—H16	118.2
C6—C1—C7	115.0 (5)	C16—C17—C18	118.7 (6)
O1—C2—C3	118.7 (6)	C16—C17—H17	120.6
O1—C2—C1	123.8 (6)	C18—C17—H17	120.6
C3—C2—C1	117.4 (6)	C19—C18—C17	120.6 (6)
C4—C3—C2	122.2 (6)	C19—C18—Cl2	121.4 (5)
C4—C3—H3	118.9	C17—C18—Cl2	118.1 (5)
C2—C3—H3	118.9	C18—C19—C14	121.1 (6)
C3—C4—C5	120.3 (6)	C18—C19—H19	119.5
C3—C4—H4	119.8	C14—C19—H19	119.5
C5—C4—H4	119.8	N2—C20—C14	127.1 (5)
C6—C5—C4	119.6 (7)	N2—C20—H20	116.5
C6—C5—Cl1	119.9 (6)	C14—C20—H20	116.5
C4—C5—Cl1	120.5 (6)	N2—C21—C22	110.4 (5)
C5—C6—C1	120.8 (6)	N2—C21—C26	109.4 (5)
C5—C6—H6	119.6	C22—C21—C26	111.4 (5)
C1—C6—H6	119.6	N2—C21—H21	108.5
N1—C7—C1	127.2 (6)	C22—C21—H21	108.5
N1—C7—H7	116.4	C26—C21—H21	108.5
C1—C7—H7	116.4	C21—C22—C23	112.4 (5)
N1—C8—C9	109.0 (5)	C21—C22—H22A	109.1
N1—C8—C13	110.7 (5)	C23—C22—H22A	109.1
C9—C8—C13	111.0 (5)	C21—C22—H22B	109.1
N1—C8—H8	108.7	C23—C22—H22B	109.1
C9—C8—H8	108.7	H22A—C22—H22B	107.9
C13—C8—H8	108.7	C24—C23—C22	111.2 (5)
C8—C9—C10	111.5 (5)	C24—C23—H23A	109.4
C8—C9—H9A	109.3	C22—C23—H23A	109.4
C10—C9—H9A	109.3	C24—C23—H23B	109.4
C8—C9—H9B	109.3	C22—C23—H23B	109.4
C10—C9—H9B	109.3	H23A—C23—H23B	108.0
H9A—C9—H9B	108.0	C23—C24—C25	110.2 (5)
C11—C10—C9	110.6 (6)	C23—C24—H24A	109.6
C11—C10—H10A	109.5	C25—C24—H24A	109.6
C9—C10—H10A	109.5	C23—C24—H24B	109.6
C11—C10—H10B	109.5	C25—C24—H24B	109.6
C9—C10—H10B	109.5	H24A—C24—H24B	108.1
H10A—C10—H10B	108.1	C26—C25—C24	111.0 (5)
C12—C11—C10	109.3 (6)	C26—C25—H25A	109.4
C12—C11—H11A	109.8	C24—C25—H25A	109.4
C10—C11—H11A	109.8	C26—C25—H25B	109.4
C12—C11—H11B	109.8	C24—C25—H25B	109.4
C10—C11—H11B	109.8	H25A—C25—H25B	108.0
H11A—C11—H11B	108.3	C25—C26—C21	111.6 (5)
C13—C12—C11	110.9 (6)	C25—C26—H26A	109.3
C13—C12—H12A	109.5	C21—C26—H26A	109.3
C11—C12—H12A	109.5	C25—C26—H26B	109.3
C13—C12—H12B	109.5	C21—C26—H26B	109.3
C11—C12—H12B	109.5	H26A—C26—H26B	108.0
